# Expression quantitative trait locus studies in the era of single-cell omics

**DOI:** 10.3389/fgene.2023.1182579

**Published:** 2023-05-22

**Authors:** Jie Luo, Xinyi Wu, Yuan Cheng, Guang Chen, Jian Wang, Xijiao Song

**Affiliations:** ^1^ State Key Laboratory for Managing Biotic and Chemical Threats to The Quality and Safety of Agro‐products, Zhejiang Academy of Agricultural Sciences, Hangzhou, China; ^2^ Institute of Vegetables, Zhejiang Academy of Agricultural Sciences, Hangzhou, China

**Keywords:** sc-eQTL, cell-type-specific, genetic variants, scRNA-seq, bulk RNA-seq

## Abstract

Genome-wide association studies have revealed that the regulation of gene expression bridges genetic variants and complex phenotypes. Profiling of the bulk transcriptome coupled with linkage analysis (expression quantitative trait locus (eQTL) mapping) has advanced our understanding of the relationship between genetic variants and gene regulation in the context of complex phenotypes. However, bulk transcriptomics has inherited limitations as the regulation of gene expression tends to be cell-type-specific. The advent of single-cell RNA-seq technology now enables the identification of the cell-type-specific regulation of gene expression through a single-cell eQTL (sc-eQTL). In this review, we first provide an overview of sc-eQTL studies, including data processing and the mapping procedure of the sc-eQTL. We then discuss the benefits and limitations of sc-eQTL analyses. Finally, we present an overview of the current and future applications of sc-eQTL discoveries.

## 1 Introduction

Over the past decades, genome-wide association studies (GWAS) have successfully identified thousands of genetic variants associated with over 100 common diseases (Visscher et al., 2017). However, the vast majority of these variants are in non-coding regions (Brodie et al., 2016) and exert their effect function by regulating gene expression. Expression quantitative trait locus (eQTL) mapping, which links genetic variants to the variation in gene expression, has largely been performed in bulk transcriptomic data generated by RNA-seq and microarray technologies. However, a significant proportion of GWAS loci cannot be explained by eQTL signals in bulk transcriptomic data, in which expression levels are averaged across all cells in a sample.

One solution to this problem is to study the regulation of gene expression at the cell-type-specific level (Knowles et al., 2017; Favé et al., 2018). Several previous studies in purified blood cell populations (Fairfax et al., 2012; Ishigaki et al., 2017; Donovan et al., 2020; Kim-Hellmuth et al., 2020; Yao et al., 2021) have already identified cell-type-specific regulation. The recent advent of scRNA-seq technology has revolutionized our ability to understand cell-type-specific gene expression by resolving complex cellular heterogeneity.

The single-cell expression quantitative trait locus (sc-eQTL) is emerging as a powerful tool to identify cell-type-specific regulation of gene expression. For example, a recent study performed eQTL mapping using single nuclei RNA-seq from 196 individuals in eight CNS cell types and identified 6,108 eGenes, 43% of which have cell-type-specific effects. The study provided new insights into the disease etiology and genetic mechanisms influencing neurological disorders (Bryois et al., 2022), demonstrating that sc-eQTL mapping provides a powerful approach to link genetic variants to complex diseases.

In this review, we aim to provide a comprehensive overview of sc-eQTL studies. We begin with an introduction to data processing and mapping procedures used in sc-eQTL analyses and provide details of the methods used in the analysis of the cell-type-specific regulation of gene expression. We then discuss the benefits of sc-eQTL studies compared to traditional eQTL analyses using bulk transcriptomic data. The limitations and challenges of sc-eQTL analyses are also discussed. Finally, we present a comprehensive overview of the current and future applications of sc-eQTL discoveries.

## 2 Evolution of sc-eQTL analyses: from an early approach to recent developments

**FIGURE 1:**
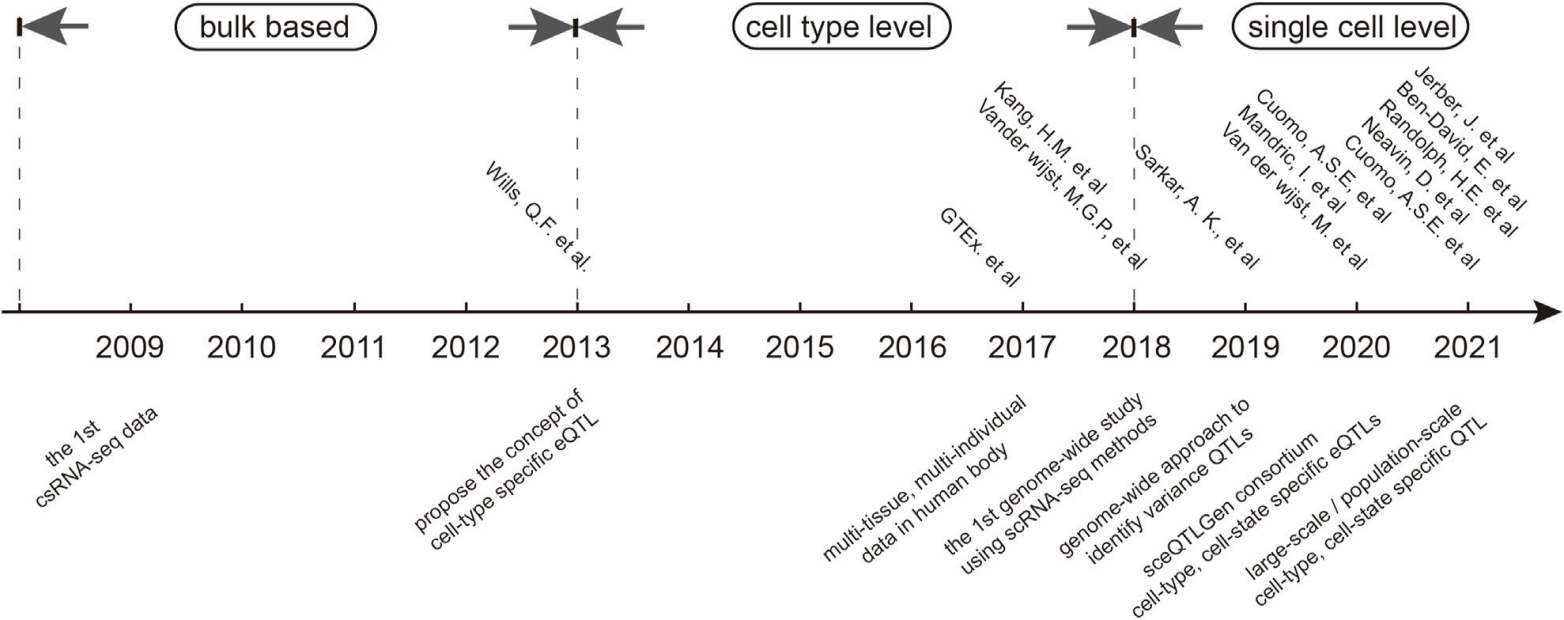
History of single-cell RNA sequencing.

The concept of cell-type-specific eQTLs was first introduced in 2013 in a study that measured 92 genes in 1,440 single cells from 15 individuals (Wills et al., 2013) to explore whether studying individual cells could provide greater mechanistic insights into how genetic variants quantitatively affect gene expression. However, the first large-scale genome-wide sc-eQTL study was performed in 2018 in eight major immune cell populations from 78,000 peripheral blood mononuclear cells (PBMCs) from 23 donors (Kang et al., 2018; Ma et al., 2022). This study was further expanded by identifying unfound cell-type-specific and co-expression eQTLs (van der Wijst et al., 2018) in 25,000 PBMCs from 45 donors. Similar sc-QTL studies using different single-cell transcriptomic technologies were also reported (Sarkar et al., 2019; Cuomo et al., 2020a; Mandric et al., 2020; Van Der Wijst et al., 2020; Figure 1). Single-cell transcriptomic technologies primarily fall into two categories: one that captures the full length of transcripts (e.g., Smart-seq2, MATQ-seq2, and SUPeR-seq) and another that captures the 3′/5′ends of transcripts. Full-length transcript sequencing allows for the detection of the complete transcriptome and the analysis of alternative splicing; its high cost and limited scalability makes it impractical for large-scale studies. In contrast, 3′/5′-end transcript sequencing, while less sensitive in detecting gene expression and alternative splicing, is more cost-effective and scalable and can, thus, accommodate more cells (Svensson et al., 2017; Chen et al., 2019). Recently, long-read sequencing technologies, such as PacBio and Oxford Nanopore, have emerged as powerful tools in the field, enabling the detection of full-length transcripts at high throughput and with high accuracy. These technologies are still in their infancy, but they hold great potential for expanding the capabilities of single-cell transcriptomic studies and can be expected to impact the sc-eQTL study.

Similar to eQTL analyses at the bulk level, gene regulation can be classified into two types: cis-regulation (local) and trans-regulation (distant). Most sc-eQTL studies have focused on cis-regulation due to the statistical power. In theory, cis-eQTLs can be mapped for all the genes measured in each cell. However, owing to the coverage of scRNA-seq, the identification of cis-eQTLs is currently only limited to cell-type levels. As a result, current sc-eQTL studies mainly attempt to identify cell-type-specific cis-eQTLs using single-cell transcriptomics (van der Wijst et al., 2018). To overcome the coverage issue of single-cell transcriptomic data and utilize expression levels measured by bulk transcriptomics, many computational deconvolution methods were developed to integrate single-cell and bulk transcriptomic data to identify cell-type-specific cis-eQTLs. However, a limitation of the deconvolution methods is that the analyzed cis-eQTLs were assigned to known cell types. Several studies also pointed out that the analysis of cis-eQTLs directly detected by single-cell transcriptomics outperforms deconvolution methods (Perez et al., 2022; Yazar et al., 2022).

## 3 Data processing for sc-eQTL mapping

While significant efforts have been made in the development of statistical methods for bulk transcriptomic data, most of these methods cannot be directly applied to sc-eQTL studies. This is because single-cell transcriptomic data have unique characteristics, such as zero-inflated gene expression. As a result, several crucial processing steps are needed to be performed before utilizing statistic methods developed for bulk RNA-seq studies on single-cell transcriptomic data.

### 3.1 Preprocessing single-cell transcriptomic data for eQTL mapping

**FIGURE 2:**
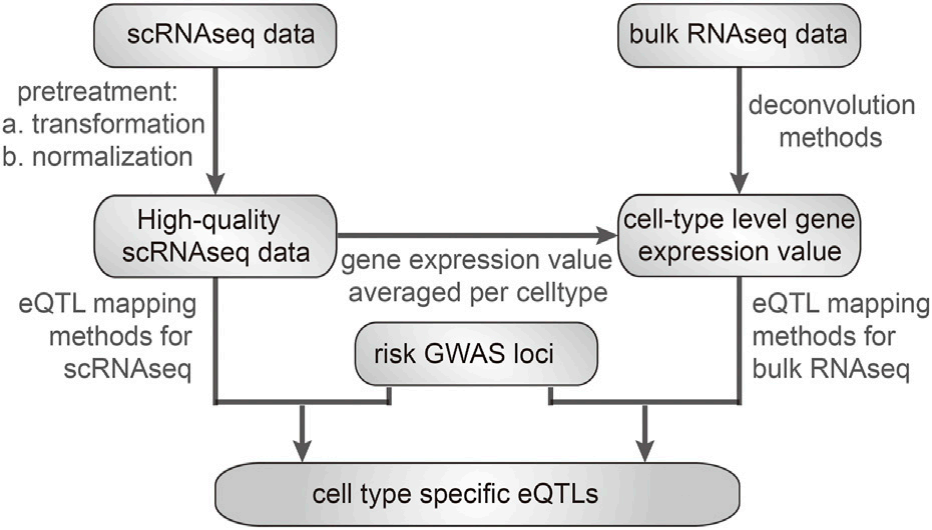
Processes for mapping cell-type-specific eQTLs.

The main processes involved in preparing single-cell transcriptomic data for eQTL mapping involve several key steps, including cell-level gene expression counting, quality control (QC), mean aggregation, covariate correlation procedures, and multiple testing corrections in the context of sc-eQTL mapping (Figure 2). A study by has provided optimized eQTL mapping workflows for single-cell studies (Cuomo et al., 2021).

The process starts with counting the cell-level gene expression, which can be obtained using a variety of different methods (Teng et al., 2016; Vieth et al., 2019; Chen et al., 2021). As for digital transcript quantification, transcripts from tag-based sequencing can be combined with UMI tags. UMI tags are a series of short sequences with specifically ordered bases; they are added to the ends of cDNAs during reverse transcription, and PCR products from the same cDNA would carry the same UMI molecule. Therefore, UMI tags can distinguish cDNA repeats from biology repeats. However, transcripts from full-length scRNA-seq cannot be combined with UMI molecules, which results in a lower quality of transcript counting based on full-length sequencing than that based on tag-based sequencing. However, MATQ-seq can produce full-length transcripts that can be combined with UMI molecules (Macosko et al., 2015).

QC steps should be performed at the cell level to remove low-quality cells and normalize data to remove technical variations in the sequencing depth per cell. Batch corrections should also be used to remove poor-quality batches. A study by (Luecken and Theis, 2019) provides an overview of their best practices. Moreover, Xue et al. (2023) proposed a new guideline to optimize the number of latent variables for bulk data batch-effect correction tools, such as probabilistic estimation of expression residuals (PEER) and principal component analysis (PCA), thereby improving the power of sc-eQTL discovery. A list of methods/tools on data transformation, scaling/normalization, and batch effect correction are provided in Table 1 and Supplementary Table S1. Among batch effect correction methods in Table 1, some are linear methods (e.g., limma and ComBat) and some belong to NN-based methods (e.g., fastMNN, Scanorama, and Seurat). The four methods (WaVE, scMerge, scVI, and LIGER) in Table 1 can handle normalization and batch correction together (Chu et al., 2022). (Tran et al., 2020) compared 14 batch effect correction methods in five scenarios. In general, the tools Harmony, LIGER, and Seurat 3 perform well in batch processing. When correcting batch effects for unknown cell types, LIGER is preferred. However, the runtime of LIGER is comparatively long. Seurat 3 enables the handling of large datasets, but requires a longer runtime. To perform downstream DEG analysis well, the scMerge tool is recommended.

**TABLE 1:** Methods/tools used for data processing in sc-eQTL mapping.

Name	Tools/package	Model/method	Reference	Site
Batch effect correction				
limma	limma	Quantitative weighting (linear-based)	Ritchie et al., 2015	http://mirrors.nju.edu.cn/bioconductor/2.11/bioc/html/limma.html
ComBat	sva	Empirical Bayesian frameworks (linear-based)	Johnson et al., 2007	http://www.bioconductor.org/packages/release/bioc/html/sva.html
MNN	scran	Mutual nearest neighbor methods (NN-based)	Haghverdi et al., 2018	https://bioconductor.org/packages/scran
BBKNN	bbknn	Fast graph-based data integration algorithm	Polański et al., 2020	https://github.com/Teichlab/bbknn
fastMNN	batchelor	(Fast version of) mutual nearest neighbor methods (NN-based)	Haghverdi et al., 2018	https://bioconductor.org/packages/release/bioc/html/scran.html
Scanorama	scanorama	NN-based	Hie et al., 2019	https://github.com/brianhie/scanorama
Seurat	Seurat	NN-based	Hao et al. (2021)	https://satijalab.org/seurat/
Harmony	harmony	Unsupervised joint embedding (linear-based)	Korsunsky et al., 2019	https://github.com/immunogenomics/harmony
scater	scater	normaliseExprs function; svaseq; RUVSeq	McCarthy et al., 2017	http://bioconductor.org/packages/scater
DCA	DCA	Negative-binomial noise model	Eraslan et al., 2019	http://github.com/theislab/dca
scGen	scGen	Variational autoencoders; latent space-vector arithmetics	Lotfollahi et al., 2019	https://github.com/theislab/scgen
Normalization and batch effect corrections together				
ZINB-WaVE	zinbwave	Extension of the RUV model	Risso et al., 2018	https://bioconductor.org/packages/zinbwave
scMerge	scMerge	MNN search and linear modeling (NN-based)	Lin et al. (2017)	https://sydneybiox.github.io/scMerge
scVI	scVI	Stochastic optimization and deep neural networks	Lopez et al., 2018	https://github.com/YosefLab/scVI
LIGER	LIGER	Integrative non-negative matrix factorization	Liu et al., 2020	https://github.com/MacoskoLab/liger

After quality control, it is necessary to perform clustering and cell-type assignment for scRNA-seq data (Cuomo et al., 2021). Major clustering tools for scRNA-seq data are based on the combination of basic clustering methods, which contain feature selection and dimensionality reduction, k-means, hierarchical clustering, and so on. Feature selection can identify genes with the highest variance. Dimensionality reduction projects data into a low-dimensional space, trying to preserve the original pairwise distances between points in the data as much as possible. Principal component analysis is one of the classical dimensionality reduction methods. Many methods, including Euclidean distance, cosine similarity, Pearson’s correlation, Spearman’s correlation, and so on, can be used to calculate the distance between points in a lower-dimensional space. K-means iteratively identifies k-cluster centers (centroids), and each cell in scRNA-seq data is assigned to the closest centroid. K-means can deal with large datasets but is not guaranteed to find the global minimum, and additionally, it is biased toward identifying equal-sized clusters, while omitting rare cell types. Another widely used clustering algorithm is hierarchical clustering, which combines individual cells into larger clusters or divides clusters into smaller groups. A visible disadvantage of hierarchical clustering is the high cost of time and memory for a large dataset. Community detection is a variant of clustering and is especially applied to graphs. This method identifies groups of nodes that are densely connected. An advantage of graph-based methods is that they do not need to specify the number of clusters.

As a single clustering method has notable disadvantages, many tools, including clustering modules, are based on a combination of several basic clustering methods. For example, clustering modules in Scanpy (Wolf et al., 2018), Seurat (Hao et al., 2021), PhenoGraph (Levine et al., 2015), SC3 (Kiselev et al., 2017; Kiselev et al., 2019), CIDR (Lin et al., 2017), pcaReduce (Žurauskienė and Yau, 2016), and TSCAN (Ji and Ji, 2016) are based on a combination of PCA and other basic clustering methods. SIMLR (Wang et al., 2018) is based on data-driven dimensionality reduction and k-means. GiniClust (Jiang et al., 2016) is based on DBSCAN; mpath and SINCERA (Guo et al., 2015) are based on hierarchical clustering; BackSPIN (Zeisel et al., 2015) is based on biclustering; RaceID3 (Grün et al., 2015) is based on k-means; and SNN-Cliq is graph-based. So, there are several user-friendly clustering tools available today. However, they have been developed for solving certain problems and it is impossible for them to be suitable for all situations.

Choosing suitable clustering and cell-type assignment algorithms for scRNA-seq data is vital (Luecken and Theis, 2019). The identification or classification of a cell into the right type or state is especially important (Van Der Wijst et al., 2020). For example, developed a clustering method based on sorting points into neighborhoods (SPIN) (Tsafrir et al., 2005). Some methods identify cell types through unsupervised clustering, such as pcaReduce and SC3. A major challenge in cell-type profiling is to identify rare cell types. A developed algorithm named rare cell-type identification (RaceID) infers abundant cell types by k-means clustering followed by systematic outlier screening (Grün et al., 2015). GiniClust detects rare cell types from single-cell gene expression data with the Gini index (Jiang et al., 2016), and GiniClust2, the upgraded version of GiniClust, is a cluster-aware weighted ensemble clustering method for cell-type detection (Tsoucas and Yuan, 2018). A newly developed tool, CellSIUS, can provide the sensitive and specific detection of rare cell populations from complex scRNA-seq data (Wegmann et al., 2019). Mean aggregation of gene expression across cells for each cell type is typically conducted by averaging gene profiles across cell types. Cell or cell-type-specific eQTLs can be mapped using eQTL mapping methods, developed especially for scRNA-seq data (Figure 2).

### 3.2 Methods used for sc-eQTL mapping

After preprocessing single-cell transcriptomic data, eQTL mapping is applied to identify genetic variants regulating gene expression at the single-cell-type level. Mapping can be carried out through various methods, including some sc-eQTL-specific tools (Table 2) and bulk eQTL mapping methods (Supplementary Table S2). These methods can be classified into two categories: parametric and non-parametric methods. Parametric methods, such as linear regression and ANOVA, assume that gene expression follows the normal distribution, Poisson distribution, or negative binomial distribution and use gene expression as the dependent variable, and genotypes as independent variables (Gatti et al., 2009; Shabalin, 2012). In contrast, non-parametric methods, such as the Krux method, are considered more robust and do not rely on any distribution assumption (Qi et al., 2014). Each tool presented in Table 2 has specific advantages. For example, SCeQTL (R package) utilizes zero-inflated negative binomial regression for eQTL mapping in scRNA-seq data (Hu et al., 2020). eQTLsingle can discover eQTLs solely through scRNA-seq data, without the use of genomic data (Ma et al., 2022). FastGxC is an efficient and powerful tool for mapping context-specific eQTLs in scRNA-seq data (Lu et al., 2021). Lastly, scTBLDA considers information across cell types, which is often ignored by methods that use summary statistics within cell types (Gewirtz et al., 2022).

**TABLE 2:** eQTL mapping methods/tools specifically for scRNA-seq data.

Tool/method	Reference	Traits	Site
SCeQTL	Hu et al. (2020)	Zero-inflated generalized linear model	https://github.com/XuegongLab/SCeQTL/
eQTLsingle	Ma et al. (2022)	Discover eQTLs only with scRNA-seq data	https://github.com/horsedayday/eQTLsingle
FastGxC	Andrew et al., 2021	Map context-specific eQTLs by leveraging the correlation structure of multi-context studies	https://github.com/BrunildaBalliu/FastGxC
scTBLDA	Gewirtz et al. (2022)	Uses MatrixEQTL v2.3 with modelLINEAR to run eQTL testing	https://github.com/gewirtz/scTBLDA

Similar to traditional bulk eQTL mapping, the effects of covariates are typically removed from a sc-eQTL analysis to improve the sensitivity and interpretability of genetic associations in population-scale expression data. For example, a recent cell-type-specific eQTL in fibroblasts and fibroblast-derived iPSC types used different covariates and probabilistic estimation of expression residual factors (Shabalin, 2012; Neavin et al., 2021). Additionally, Xue et al. (2023) highlighted three key differences between bulk data and scRNA-seq pseudo-bulk data and provided a new guideline for selecting the optimal number of latent variables for bulk data batch-effect correction tools. This guideline has the potential to significantly improve sc-eQTL discovery and is an important contribution to the field.

The method specifically developed for sc-eQTL mapping can efficiently identify context-specific genetic variants regulating gene expression at the cell-type-specific level. For example, a method called FastGxC enables the construction of context-specific eQTL maps and has the potential to increase precision in identifying GWAS variants by three-fold compared to conventional eQTL mapping methods (Lu et al., 2021).

Compared to conventional eQTL mapping methods, sc-eQTL mapping strategies face the challenge of excessive zeros in single-cell transcriptomic data (Delmans and Hemberg, 2016; Miao et al., 2018; Hu et al., 2020). To address this challenge, the R package SCeQTL uses zero-inflated negative binomial regression for the sc-eQTL analysis to detect the gene expression variation and distinguish between “status difference” and “expression level difference” (Hu et al., 2020). Some recent approaches also take into account the dynamic pseudotime-defined cell types for the sc-eQTL analysis (Cuomo et al., 2020b), which have been shown to uncover new eQTL variants. In addition, the eQTLsingle tool was developed to discover eQTLs solely with single-cell transcriptomic data and detect mutations from single-cell transcriptomic data as genotypic data (Ma et al., 2022).

## 4 Advantages and limitations of sc-eQTL mapping

### 4.1 Advantages of sc-eQTL mapping compared to bulk eQTL methods

**FIGURE 3:**
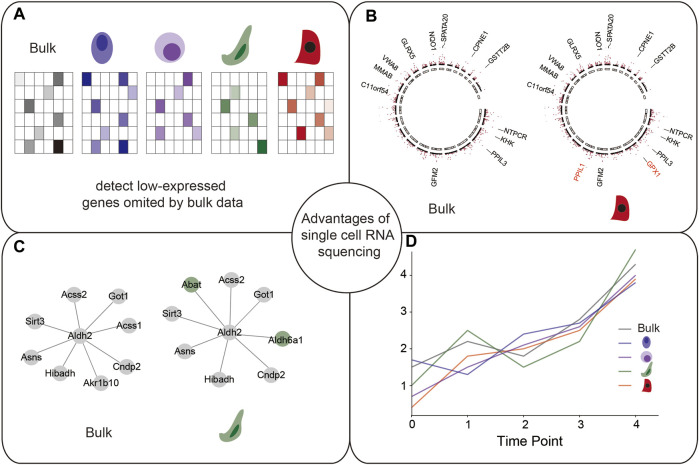
Advantages of scRNA-seq data, including **(A)** Identifying cell-type-specific eQTLs; **(B)** identifying low-expressed genes; **(C)** identifying cell-type-specific co-expression networks; and **(D)** identifying cell-type-specific eQTLs in different spatiotemporal states.

Single-cell transcriptomic data provide several advantages in exploring the genetic architecture of gene regulation. The ability of detecting cell types and cell states in an unbiased manner using single-cell transcriptomic data makes sc-eQTL mapping a powerful tool for studying the genetic architecture of gene regulation (Grün et al., 2015; Villani et al., 2017; Hernández et al., 2018; Karamitros et al., 2018; Guerrero-Juarez et al., 2019; Umans et al., 2020).The advantages of sc-eQTL mapping include the following: 1) discovery of cell-type-specific eQTLs, 2) identification of eQTLs regulating lowly expressed genes, and 3) detection cell-type-specific eQTLs in different spatiotemporal states. We discuss these advantages in detail in the following sections (Figure 3).

#### 4.1.1 Discovery of cell-type-specific eQTLs that are diluted in bulk RNA-seq

Single-cell transcriptomic data offer a powerful tool to uncover cell-type-specific eQTLs that are diluted in bulk transcriptomic data. Cell-type-specific cis-eQTLs identified by bulk RNA-seq data are biased to known cell types, while the ones identified by scRNA-seq data can be assigned to novel cell types. Multiple studies have demonstrated this advantage. For example, a study discovered 379 cis-eQTLs (287 genes), of which 48 cis-eQTLs (38 genes) were only detected in specific cell types, not in any eQTLs from bulk RNA-seq data (van der Wijst et al., 2018). Another study on human skin fibroblasts showed that a majority of discovered eGenes were predominantly cell-type-specific and could only be identified in one fibroblast type or one iPSC type (Neavin et al., 2021). These findings suggest a high degree of cell-type-specific gene regulations detected in the sc-eQTL analysis that cannot be captured by bulk QTL mapping. Hence, sc-eQTL can be used to improve the eQTL detection when compared to bulk RNA-seq.

#### 4.1.2 Identification of eQTLs regulating lowly expressed genes that are omitted by bulk data

Compared with bulk RNA-seq data, scRNA-seq data allow the estimation of the variability in gene expression across individual cells (Brennecke et al., 2013) and provide a new angle on how genetics may impact disease pathogenesis. For example, owing to the low expression of TSPAN13 in abundant CD4^+^ T cells, cis-eQTL rs2272245 was not identified in the bulk RNA-seq dataset (Zhernakova et al., 2017), but it significantly affected the low expressed gene TSPAN13 in cis (*p* = 2.21 × 10^−6^) in the scRNA-seq data analysis. This shows that the bulk RNA-seq-based cis-eQTL analysis loses power in the identification of cell-type-specific loci affecting lowly expressed genes (van der Wijst et al., 2018).

#### 4.1.3 Detection of cell-state-specific eQTLs while bulk data lose this power

scRNA-seq data enable the simultaneous estimation of the composition and expression profiles of discrete cell populations, such as their activation states (van der Wijst et al., 2018). scRNA-seq data provide a flexible unbiased approach that has increased their resolution to define cell states along continuous dynamic processes, in which the eQTL effects manifest themselves (Cuomo et al., 2020a). In an elegant study by, the authors derived 126 iPSC cell lines from 125 donors in the HipSci project (Kilpinen et al., 2017) and harvested the cells immediately before differentiation (iPSCs) and at the mesendoderm and definitive endoderm stage of differentiation (Cuomo et al., 2020b). They found that over 30% of the identified eQTLs were specific to a single stage. Moreover, 349 eQTL variants identified during differentiation stages were novel and not previously identified in bulk RNA-Seq from iPSCs or GTEx tissues, and they also illustrated that eQTLs can modulate the timing of expression changes in response to differentiation (Cuomo et al., 2020a). Altogether the study demonstrated that the identification of eQTLs at distinct time points in the development allows the discovery of novel regulatory relationships.

In a study by the mapped eQTLs in memory T cells from 259 Peruvian individuals revealed more than 2,000 eQTLs, whose presence and function varied according to the transcriptomic state of T cells. So, they demonstrated that DNA sequence variation at a particular location in the genome may influence the expression of a given gene in some T-cell states but not in others (Nathan et al., 2022).

Another study by Yazar et al. (2022) identified cell-state-dependent eQTLs in B cells transitioning from naïve to memory states. In an example with rs9927852 and MAF, the expression of MAF increased with a high cytotoxic cell-state score and remained relatively constant with low cell-state scores. So, they demonstrated that two independent eQTLs have opposite effects on the expression of the same gene in different cell states. The above two studies emphasize the complexity of genome regulation in immune cells, and scRNA-seq increases the resolution of the identified eQTLs (Yazar and Powell, 2022).

### 4.2 Limitations of scRNA-seq in eQTL mapping

Despite the many benefits of sc-eQTL mapping, as shown previously, several limitations have also been noted in recent studies. These limitations include the following: 1) less power in identifying eQTLs, 2) high cost of scRNA sequencing, and 3) technical noises in scRNA-seq data.

#### 4.2.1 Less power in identifying eQTLs

sc-eQTL mapping provides a detailed annotation of the eQTL effects across diverse cell types and cell states, enabling a better interpretation of the context-specific role of individual genetic variants (Cuomo et al., 2020b). However, owing to increased experimental noise, sc-eQTL mapping has lower power to discover eQTLs compared to bulk RNA-seq data. Thus, scRNA-seq data require larger sample sizes to identify the same number of eQTLs as bulk data (Sarkar et al., 2019). For instance, scRNA-seq studies by and Perez et al., 2022 identified less than 15 cell types, whereas Ota et al., 2021 identified 28 cell types in bulk RNA-seq data (Ota et al., 2021; Perez et al., 2022; Yazar et al., 2022). As a result, if the same sample size is used for scRNA-seq, a lower number of cis-eQTLs will be detected in scRNA-seq data compared to bulk data.

#### 4.2.2 High cost of scRNA sequencing

The second limitation of the sc-eQTL study is the high cost associated with scRNA-seq, which is a relatively expensive method for gene expression analysis. While a typical bulk RNA-sequencing experiment requires up to 20 million sequencing reads per sample, scRNA-seq needs a much higher coverage, typically 50,000 to 150,000 reads per cell. A simple scRNA-seq experiment would include thousands of cells, with hundreds of thousands of reads. For example, to detect one thousand reads per cell, it needs to detect 50–150 million reads per sample, where the number of reads captured in scRNA-seq is 2.5–7.5 times larger than that in bulk RNA-seq. Therefore, scRNA-seq needs much more memory and storage space than bulk RNA-seq experiments.

#### 4.2.3 Noise in the scRNA-seq dataset

scRNA-seq data are high dimensional and complex. When compared to traditional bulk RNA-seq, scRNA-seq needs to amplify genetic material in each cell to meet the requirements of sequencing platforms. The amplification processes bring many technical problems, such as a notable amplification bias and low genome coverage in DNA amplification, so the clustering and homogenization analysis strategies used in bulk RNA-seq cannot be used directly in scRNA-seq data analyses. As a result, there are many differences in various cells and platforms, and library sizes vary greatly between each other. So, there is much more noises in scRNA-seq data, which demand a series of pretreatment steps before the scRNA-seq data analysis.

### 4.3 Strategies to overcome the limitations of scRNA-seq in mapping eQTLs

#### 4.3.1 Decreasing the cost of scRNA-seq

One of the main limitations of scRNA-seq is its high cost. However, with the development of cost-effective multiplexed workflows, that limitation has been significantly mitigated, enabling a broader adoption of population-scale scRNA-seq and cell-type-specific eQTL studies (van der Wijst et al., 2018; Zhang et al., 2018; Cuomo et al., 2020a). Through a series of simulations, Igor M. et al. demonstrated that by increasing the sample size and number of cells per individual while decreasing coverage, it was possible to reduce the cost of the scRNA-seq experiment by half (or even more), while maintaining the same statistical power. Furthermore, they provided a practical guideline for designing cell-type-specific eQTLs (Mandric et al., 2020).

#### 4.3.2 Developing methods for deconvoluting bulk RNA-seq signals into different cell types

The high cost of single-cell transcriptomic sequencing has led to the development of several deconvolution methods to estimate the cell-type level gene expression from the bulk mRNA expression. These deconvolution methods, such as DeconRNAseq (Gong and Szustakowski, 2013), CIBERSORT (Newman et al., 2015), CIBERSORTx (Newman et al., 2019), BSEQ-sc (Baron et al., 2016), TIMER (Li et al., 2016), MuSiC (Qin et al., 2021), DSA (Zhong et al., 2013), and MMAD (Liebner et al., 2014), have been compared and discussed in recent literature (Avila Cobos et al., 2020; Jin and Liu, 2020). For instance, CIBERSORTx extends CIBERSORT to infer cell-type-specific gene expression profiles without physical cell isolation. Detailed information on the deconvolution methods is listed in Table 3. These tools are highly useful in re-analyzing both existing and new bulk RNA-seq datasets to identify and interpret the role of cell-type-specific eQTLs in complex diseases. The most widely used bulk deconvolution methods (i.e., OLS, nnls, RLR, FARDEEP, and CIBERSORT) and the three methods that use the scRNA-seq data as a reference (i.e., DWLS, MuSiC, and SCDC) achieved median RMSE values lower than 0.05 (Avila Cobos et al., 2020).

**TABLE 3:** Computational deconvolution methods.

Name	Deconvolution model	Site
Methods without scRNA-seq data as a reference		
OLS	Least squares	https://link.springer.com/chapter/10.1007/978-3-642–50096-1_48
nnls	Least squares	https://cran.r-project.org/web/packages/nnls/index.html
FARDEEP	Robust regression	https://CRAN.R-project.org/package = FARDEEP
RLR	Robust regression	https://CRAN.R-project.org/package = MASS
LASSO	Penalized regression	http://xai-tools.drwhy.ai/glmnet.html
Ridge	Penalized regression	http://xai-tools.drwhy.ai/glmnet.html
Elastic net	Penalized regression	http://xai-tools.drwhy.ai/glmnet.html
DCQ	Penalized regression	http://dcq.tau.ac.il/
EPIC	Weighted least squares	http://epic.gfellerlab.org/
CIBERSORT	Support-vector regression	http://cibersort.stanford.edu/
dtangle	Model in the logarithmic scale	dtangle.github.io
DSA	Digital sorting algorithm	http://web.cbio.uct.ac.za/∼renaud/CRAN/web/CellMix
ssKL	Semi-supervised non-negative matrix factorization	http://web.cbio.uct.ac.za/∼renaud/CRAN/web/CellMix
ssFrobenius	Semi-supervised non-negative matrix factorization	http://web.cbio.uct.ac.za/∼renaud/CRAN/web/CellMix
DeconRNASeq	Quadratic programming	https://bioconductor.org/packages/DeconRNASeq/
TIMER	Monte Carlo simulation; pathological approach	http://cistrome.org/TIMER
**Methods with scRNA-seq data as reference**		
Bisque	Regression-based approach	https://github.com/cozygene/bisque
deconvSeq	Generalized linear model	https://github.com/rosedu1/deconvSeq
DWLS	Weighted least squares	https://github.com/sistia01/DWLS
MuSiC	Weighted non-negative least squares regression (W-NNLS)	https://github.com/xuranw/MuSiC
SCDC	ENSEMBLE method	http://meichendong.github.io/SCDC
BSEQ-sc	csSAM methodology	http://github.com/shenorrlab/bseq-sc
CIBERSORTx	Support vector	https://cibersortx.stanford.edu/

Detailed information for convolution methods in Table 3: OLS (ordinary least squares (Chambers et al., 1990)), NNLS (non-negative least squares (Mullen and Stokkum, 2012)), FARDEEP (Fast And Robust DEconvolution of Expression Profiles (Hao et al., 2019)), RLR (robust linear regression, MASS (Ripley et al., 2022)), LASSO (in glmnet (Friedman et al., 2010)), Ridge (in glmnet (Friedman et al., 2010)), Elastic net (in glmnet (Friedman et al., 2010)), DCQ (digital cell quantifier (Altboum et al., 2014)), DSA (digital sorting algorithm, in CellMix (Gaujoux and Seoighe, 2013)), ssKL (in CellMix (Gaujoux and Seoighe, 2013)), ssFrobenius (in CellMix (Gaujoux and Seoighe, 2013)), EPIC (estimating the proportion of immune and cancer cells (Racle et al., 2017)), CIBERSORT (cell-type identification by estimating relative subsets of RNA transcripts (Newman et al., 2015)), dtangle ((Hunt et al., 2019)), DeconRNASeq ((Gaujoux and Seoighe, 2013)), TIMER (Tumor IMmune Estimation Resource (Li et al., 2016)), Bisque ((Jew et al., 2020)), deconvSeq ((Du et al., 2019)), DWLS (dampened weighted-least squares (Tsoucas et al., 2019)), MuSiC (multi-subject single cell (Wang et al., 2019)), SCDC ((Dong et al., 2021)), BSEQ-sc (bulk sequence single-cell (Baron et al., 2016)), CIBERSORTx ((Newman et al., 2019)).

#### 4.3.3 Batch effect correction and normalization to reduce high technical noise in scRNA-seq

Reducing high technical noise in scRNA-seq data remains a challenge. The noise can arise from differences in the sequencing platform, sequencing depth, amplification bias, RNA capture efficiency, and dropout events. Current noise reduction methods for scRNA-seq data include correcting the batch effect and normalization of the sequencing data. Recently, a comprehensive study evaluated 28 noise reduction methods and tools using 55 real and simulated datasets (Chu et al., 2022). However, it was noted that no single method can be used for all scRNA-seq experiments. The advantages and pitfalls of typical methods for batch effect correction and normalization are listed in Table 4. Therefore, the selection of an appropriate method needs caution and depends on the study design. Additionally, increasing the sample size is a feasible strategy for reducing experimental noise in scRNA-seq.

**TABLE 4:** Advantages and pitfalls of typical methods for batch effect correction and normalization.

Method	Advantages	Pitfalls
ComBat	Corrects for known and unknown batch effects	May not work well with highly variable genes
fastMNN	Handles analysis between two datasets and better accuracy	Lacks explainability
Seurat 3	Integrated with clustering and downstream analyses	May introduce unwanted sources of variation
Harmony	Corrects for batch effects while preserving biological signal	Requires careful selection of parameters
scMerge	Handles batch effects and integrates data from multiple batches	Performance may depend on the number of clusters in each batch
LIGER	Handles batch effects and normalization for unknown cell types	Requires a comparatively long runtime

## 5 Conclusion and future directions

In conclusion, this review provided an overview of the recent advances in the study of the genetic regulation of gene expression through single-cell eQTL mapping. We also discussed how to perform sc-eQTL mapping and the advantages of scRNA-seq for sc-eQTL mapping and its challenges and limitations. While sc-eQTL analysis is still in its infancy stage, it offers great potential for advancing our understanding of the genetic regulation of gene expression.

In future, the advent of single-cell transcriptomics will lead to significant advancements in the understanding of the genetic regulation of gene expression. sc-eQTL studies have revealed many previously undetected cell-type-specific eQTLs that provide new insights into disease biology. With the decrease in single-cell transcriptomic sequencing costs, sc-eQTL studies will identify new genetic variants that regulate gene expression. Furthermore, the integration of QTL signals from multi-omics at the single-cell level and spatial data can improve the resolution of gene regulation at different omics levels.
