# Supplementary material for: Expression quantitative trait locus studies in the era of single-cell omics
Source: Front Genet. 2023 May 22;14:1182579. doi: 10.3389/fgene.2023.1182579 (PMC10239882; doi:10.3389/fgene.2023.1182579)
Supplement: Supplementary file 1 [file Table1.DOCX]

Supplementary Material

**Expression quantitative trait locus (eQTL) studies in the era of single-cell omics**

Jie Luo^1*^, Xinyi Wu^2^, Yuan Cheng^2^, Guang Chen^1^, Jian Wang^1^, Xijiao Song^1^

^1^State Key Laboratory for Managing Biotic and Chemical Threats to the Quality and Safety of Agro‐products, Zhejiang Academy of Agricultural Sciences, Hangzhou, Zhejiang 310021, China

^2^Institute of Vegetables, Zhejiang Academy of Agricultural Sciences, Hangzhou, China

*** Correspondence:**

Jie Luo

luojie@mail.zaas.ac.cn

# Supplementary Tables

**Supplementary Table 1. Transformation, scaling/normalization methods for scRNA-seq data pretreatment**

| **Name** | **Tools/Package** | **Model/Method** | **Reference** | **Site** |
| --- | --- | --- | --- | --- |
| **Transformation** | | | | |
| Linear | sctransform | generalized linear model | Hafemeister C et al., 2019 | github.com/ChristophH/sctransform |
| Log | -- | -- | -- | -- |
| Sqrt | -- | -- | -- | -- |
| VST | DESeq | variance-stabilizing transformation | Anders et al., 2010 | http://www.bioconductor.org/help/search/index.html?q=DESeq/ |
| PCA | prcomp function | -- | -- | -- |
| **Scaling/Normalization** | | | | |
| Scran | scran | utilizing the scaling factor | Lun A.T.L et al., 2016 | http://bioconductor.org/packages/scran |
| SCnorm | SCnorm | utilizing the scaling factor | Bacher R et al., 2017 | https://www.biostat.wisc.edu/~kendzior/SCNORM/ |
| sctransform | sctransform | generalized linear model | Hafemeister C et al., 2019 | github.com/ChristophH/sctransform |
| QN | Affy | quantile normalization method | Bolstad BM et al., 2002 | http://www.bioconductor.org/packages/release/bioc/html/affy.html |
| UQ | Genominator; GenomeGraphs | quantile based | Bullard JH et al., 2010 | http://bioconductor.org/packages/release/bioc/html/Genominator.html; http://bioconductor.org/packages/release/bioc/html/GenomeGraphs.html |
| TPM | RSEM | generative model; EM algorithm | Li B et al., 2010 | http://deweylab.biostat.wisc.edu/rsem |
| TMM | edgeR | weighted trimmed mean of the log expression ratios | Robinson MD et al., 2010 | http://www.bioconductor.org/packages/release/bioc/html/edgeR.html |
| Median of ratios | DESeq | negative binomial distribution based | Anders et al., 2010 | http://www.bioconductor.org/help/search/index.html?q=DESeq/ |
| LogNormalize | Seurat/Scran/SCnorm | Normalize count data per cell and transform to log scale | LogNormalize function \| R Documentation | <https://www.rdocumentation.org/packages/Seurat/versions/3.1.1/topics/LogNormalize> |
| Linnorm | Linnorm | linear model | Shun HY et al., 2017 | <https://www.bioconductor.org/packages/release/bioc/html/Linnorm.html> |
| scater | scater | trimmed mean of M vanlues; relative log-expression; upper-quartile methods et al. | McCarthy DJ et al., 2017 | http://bioconductor.org/packages/scater |
| SAMstrt | SAMstrt | Poisson resampling; non-parametric statistics; scaling factor | Shintaro K et al., 2013 | https://github.com/shka/R-SAMstrt |
| BASiCS | BASiCS | integrated Bayes-ian hierarchical model | Vallejos CA et al., 2015 | https://github.com/catavallejos/BASiCS |

Full Name of methods in Table 1 and Table S1: QN (Quantile normalization); UQ(Upper quartile); TPM(Transcripts per million); TMM(Trimmed mean of M-values); BASiCS(Bayesian Analysis of Single-Cell Sequencing data); MNN (mutual nearest neighbors); BBKNN (Batch balanced KNN); fastMNN (mutual nearest neighbors); DCA (deep count autoencoder network); ZINBWaVE (Zero-Inflated Negative Binomial-based Wanted Variation Extraction); scVI (single-cell variational inference); LIGER (linked inference of genomic experimental relationships).

**Supplementary Table 2. Bulk eQTL mapping methods that are also suitable for scRNA-seq data analysis.**

| Tool/Method | Reference | Traits | Site |
| --- | --- | --- | --- |
| linear regression model | Drew Neavin et al., 2021 | can modify covariates and PEER factores in linear models | -- |
| LMM(linear mixed model) | -- | have flexibilities and abilities to robustly control for confounding factors | -- |
| Matrix eQTL | Shabalin AA et al, 2012 | supports additive linear and ANOVA models with covariates, including models with correlated and heteroskedastic errors. | http://www.bios.unc.edu/research/genomic_software/Matrix_eQTL |
| QTLtools | Delaneau O et al., 2017 | a modular framework to discover proximal and distal molQTLs, integrate molQTLs with GWAS variants and so on | <https://qtltools.github.io/qtltools/> |
| LIMIX_QTL | Cuomo ASE et al., 2021 | using linear mixed model (LMM) to map eQTLs | <https://github.com/single-cell-genetics/limix_qtl> |
| FastMap | Gatti DM et al., 2008 | use a Hamming distance-based tree to organize SNPs to map eQTLs | http://cebc.unc.edu/fastmap86.html |
| kruX | Qi jl et al., 2014 | use of robust non-parametric methods for massive eQTL mapping | [http://krux.googlecode.com](http://krux.googlecode.com/) |
| FastQTL | Ongen H et al., 2016 | improves on Matrix eQTL, by adding permutation scheme modeled by using a beta distribution | <http://fastqtl.sourceforge.net/> |
